# GeXP analyzer-based multiplex reverse-transcription PCR assay for the simultaneous detection and differentiation of eleven duck viruses

**DOI:** 10.1186/s12866-015-0590-6

**Published:** 2015-10-30

**Authors:** Yan-fang Zhang, Zhi-xun Xie, Li-ji Xie, Xian-wen Deng, Zhi-qin Xie, Si-si Luo, Li Huang, Jiao-ling Huang, Ting-ting Zeng

**Affiliations:** Department of Biotechnology, Guangxi Key Laboratory of Animal Vaccines and Diagnostics, Guangxi Veterinary Research Institute, 51 Youai North Road, Nanning, 530001 China

**Keywords:** Duck virus, Genome Lab Gene Expression Profiler (GeXP), Multiplex PCR, Separation identification

## Abstract

**Background:**

Duck viral pathogens primarily include the avian influenza virus (AIV) subtypes H5, H7, and H9; duck hepatitis virus (DHV); duck tembusu virus (DTMUV); egg drop syndrome virus (EDSV); duck enteritis virus (DEV); Newcastle disease virus (NDV); duck circovirus (DuCV); muscovy duck reovirus (MDRV); and muscovy duck parvovirus (MDPV). These pathogens cause great economic losses to China’s duck breeding industry.

**Result:**

A rapid, specific, sensitive and high-throughput GeXP-based multiplex PCR assay consisting of chimeric primer-based PCR amplification with fluorescent labeling and capillary electrophoresis separation was developed and optimized to simultaneously detect these eleven viral pathogens. Single and mixed pathogen cDNA/DNA templates were used to evaluate the specificity of the GeXP-multiplex assay. Corresponding specific DNA products were amplified from each pathogen. Other pathogens, including duck *Escherichia coli*, duck *Salmonella,* duck *Staphylococcus aureus, Pasteurella multocida,* infectious bronchitis virus, and *Mycoplasma gallisepticum*, did not result in amplification products. The detection limit of GeXP was 10^3^copies when all twelve pre-mixed plasmids containing the target genes of eleven types of duck viruses were present. To further evaluate the reliability of GeXP, 150 clinical field samples were evaluated. Comparison with the results of conventional PCR methods for the field samples, the GeXP-multiplex PCR method was more sensitive and accurate.

**Conclusion:**

This GeXP-based multiplex PCR method can be utilized for the rapid differential diagnosis of clinical samples as an effective tool to prevent and control duck viruses with similar clinical symptoms.

## Background

According to the Food and Agriculture Organization (FAO) statistics, the 771 million ducks annually raised for meat purposes in China accounted for 65.73 % of the world’s stock in 2009 (FAO, 2009). There are eleven important viral pathogens that cause infections in ducks in China, including the avian influenza virus (AIV) subtypes H5, H7, H9; duck hepatitis virus (DHV); duck tembusu virus (DTMUV); egg drop syndrome virus (EDSV); duck enteritis virus (DEV); Newcastle disease virus (NDV); duck circovirus (DuCV); muscovy duck reovirus (MDRV); and muscovy duck parvovirus (MDPV) [1–4]. With the development of the commercial duck industry in China and in other parts of the world in recent years, the incidence of duck diseases has increased [5].

The increase in duck disease has become an important factor that restricts the growth and further development of the duck industry. Differential diagnosis of infectious duck diseases using traditional methods requires isolation of the pathogens and identification using serological techniques [6–9]. The accuracy of these methods is often affected by the freshness of the clinical material, contamination with bacteria and the length of time between the collection of the material and its analysis. Thus conventional methods of diagnosis are very tedious and time-consuming and are further complicated by the differential diagnosis of mixed infections [10, 11]. Molecular typing methods for the rapid detection and identification of pathogens have been developed and are used currently. Although useful, most molecular methods are limited to the detection of a few pathogens in one reaction [12–16]. Therefore, a rapid, cost-effective and high-throughput method for the detection and differentiation of viral pathogens in one test tube would be advantageous and would ensure prompt treatment and infection control.

The GenomeLab Gene Expression Profiler (GeXP) analyzer is a multiplex gene expression analysis platform developed by Beckman Coulter (Brea, CA, USA). GeXP assays have been successfully used for rapid identification in human medicine including assays to detect several inflammatory and cytokine gene targets in normal colon tissue as well as in colon polyps and tumors [17, 18], prostate cancer biomarker gene expression signatures in biological samples [19], pandemic influenza A H1N1 virus [20]; nine types of enteroviruses associated with hand, foot, and mouth disease [21]; and eleven human papillomaviruses in a rapid and sensitive manner [22]. Recently, a GeXP assay was used for the detection of nine avian respiratory disease agents [23]. The GeXP analyzer has a built-in software program that can be used to evaluate PCR products based on amplicon size. The software displays the differences between the various specific genes under investigation and compares the expected PCR product sizes to identify each product. The GeXP-multiplex PCR assay provide high sensitivity and specificity compared to other multiple-detection methods [24].

In this study, a GeXP analyzer-based multiplex RT-PCR assay (GeXP-multiplex PCR) was developed to simultaneously detect eleven common duck viral diseases in China, including AIV-H5, AIV-H7, AIV-H9, DHV, DTMUV, EDSV, DEV, NDV, DuCV, MDRV, and MDPV. The diagnostic sensitivities and specificities of this assay were evaluated with 150 clinical specimens.

## Methods

### Ethics statement

This study was approved by the Institutional Animal Care and Use Committee (IACUC) of the Guangxi Veterinary Research Institute. Some specimens were collected from ducks that had died from disease. The other biological samples were briefly and gently collected from the larynx, trachea and cloaca of healthy ducks using sterilized cotton swabs without anesthesia after receiving the verbal permission of the owners’ verbal permission. The sampled ducks were observed for 30 min after sampling and were then returned to their cages.

### Extraction of DNA/RNA from pathogens and sample preparation

The avian pathogen reference strains, field isolates and other duck pathogens used in this study are described in Table 1. Viral RNA and DNA were extracted using the MiniBEST Viral RNA/DNA Extraction Kit Ver5.0 (Takara, Dalian, China) according to the manufacturer’s protocol. The DNA and RNA samples were aliquoted and stored at −30 °C until use.

**Table 1:** Sources of pathogens used and GeXP assay results

Pathogen/field samples	Number of samples	Source	Results											
			AIV- M	AIV- H5	AIV- H7	AIV- H9	DHV	DEV	DTMUV	NDV	EDSV	MDRV	MDPV	DuCV
Reference sample														
^a^cDNA of H5N3 AIV Duck/HK 313/78	1	HKU	+	+	−	−	−	−	−	−	−	−	−	−
^a^H7N2 AIV Duck/HK/ 47/76	1	HKU	+	−	+	−	−	−	−	−	−	−	−	−
^a^H9N6 AIV Duck/HK/147/77	1	HKU	+	−	−	+	−	−	−	−	−	−	−	−
^b^DHV (AV2111)	1	CIVDC	−	−	−	−	+	−	−	−	−	−	−	−
^c^DEV (AV1221)	1	CIVDC	−	−	−	−	−	+	−	−	−	−	−	−
^d^DTMUV (GX201301,GX201302)	2	GVRI	−	−	−	−	−	−	+	−	−	−	−	−
^e^NDV (GX1/00,GX6/02)	2	GVRI	−	−	−	−	−	−	−	+	−	−	−	−
^f^EDSV (GEV)	1	GVRI	−	−	−	−	−	−	−	−	+	−	−	−
^g^MDRV (NM1,NM2)	2	GVRI	−	−	−	−	−	−	−	−	−	+	−	−
^h^MDPV (GX-5,GX-6)	2	GVRI	−	−	−	−	−	−	−	−	−	−	+	
^i^DuCV (GX1006,GX1008)	2	GVRI	−	−	−	−	−	−	−	−	−	−	−	+
Other pathogens														
H1N3 AIVDuck/HK/717/79-d1	1	HKU	+	−	−	−	−	−	−	−	−	−	−	−
H1N1 AIV Human/NJ/8/76	1	HKU	+	−	−	−	−	−	−	−	−	−	−	−
H2N3 AIV Duck/HK/77/76	1	HKU	+	−	−	−	−	−	−	−	−	−	−	−
H3N6 AIV Duck/HK/526/79/2B	1	HKU	+	−	−	−	−	−	−	−	−	−	−	−
H4N5 AIV Duck/HK/668/79	1	HKU	+	−	−	−	−	−	−	−	−	−	−	−
H6N8 AIV Duck/HK/531/79	1	HKU	+	−	−	−	−	−	−	−	−	−	−	−
H8N4AIV Turkey/ont/6118/68	1	HKU	+	−	−	−	−	−	−	−	−	−	−	−
H10N3 AIV Duck/HK/876/80	1	HKU	+	−	−	−	−	−	−	−	−	−	−	−
H11N3 AIV Duck/HK/661/79	1	HKU	+	−	−	−	−	−	−	−	−	−	−	−
H12N5 AIV Duck/HK/862/80	1	HKU	+	−	−	−	−	−	−	−	−	−	−	−
H6N8 AIV Duck/HK/531/79	1	HKU	+	−	−	−	−	−	−	−	−	−	−	−
duck *Escherichia coli*	1	GVRI	−	−	−	−	−	−	−	−	−	−	−	−
duck *Salmonella*	1	GVRI	−	−	−	−	−	−	−	−	−	−	−	−
duck *Staphylococcus aureus*	1	GVRI	−	−	−	−	−	−	−	−	−	−	−	−
Pasteurella multocida	1	GVRI	−	−	−	−	−	−	−	−	−	−	−	−
Infectious bronchitis virus	1	GVRI	−	−	−	−	−	−	−	−	−	−	−	−
Mycoplasma gallisepticum	1	GVRI	−	−	−	−	−	−	−	−	−	−	−	−
Sample mixture														
AIV-H5 + AIV−H7 + AIV−H9	1	GVRI	+	+	+	+	−	−	−	−	−	−	−	−
AIV−H5 + DEV + DTMUV + NDV + EDSV	1	GVRI	+	+	−	−	−	+	+	+	+	−	−	−
AIV−H5 + AIV−H7 + AIV−H9 + DHV + DEV + DTMUV + NDV + EDSV+ MDRV+ MDPV+ DuCV	1	GVRI	+	+	+	+	+	+	+	+	+	+	+	+

*HVRI* Harbin Veterinary Research Institute, China, *HKU* The University of HongKong, China, *GVRI* Guangxi Veterinary Research Institute, China, *CIVDC* China Institute of Veterinary Drug Control, China, *PU* University of Pennsylvania

^a^References [23]

^b^GenBank accession no. : EF442073.1

^c^GenBank accession no. : EU315247

^d^GenBank accession no. : KJ700462.1

^e^GenBank accession no. : JX193083.1

^f^References [35]

^g^References [35]

^h^GenBank accession no. : KM093740.1

^i^GenBank accession no. : JX241046.1

### Primer design and plasmid preparation

The GeXP-multiplex assay consists of twelve pairs of chimeric primers including one pair of AIV universal primers (AIV *M*) and eleven pairs of duck virus primers. These twelve pairs of duck virus primers were designed based on the sequences of the type A AIV *M* gene; the *HA* genes from the H5 and H7 and H9 subtypes of AIV; the *5′UTR* region of the DHV gene; the DTMUV *E* gene; the DEV *UL6* gene; the NDV *L* gene; the EDSV *Penton* gene; the MDRV *S1* gene; the MDPV *VP1* gene and the DuCV *Red* gene. All sequences were obtained from GenBank. The primers were designed by selecting the highly conserved regions using DNAstar software (DNASTAR Inc., Madison, WI, USA) and Primer Premier 5.0 software (Premier Biosoft International, Palo Alto, CA, USA). AIV universal primers were utilized to amplify all 16 subtypes. The 5′ end of the forward universal primer (Tag-F: AGGTGACACTATAGAATA) was labeled and purified with high-pressure liquid chromatography. All chimeric primers and the reverse universal primer (Tag-R: GTACGACTCACTATAGGGA) were synthesized and purified by polyacrylamide gel electrophoresis (BGI, China). The primer sequences, the size of the resulting amplicons, and the target regions are listed in Table 2.

**Table 2:** Primers used in this study

Virus	Forward primer sequence (5′−3′)	Reverse primer sequence (5′−3′)	Amplicon size (bp)	Target region
**AIV-M**	**AGGTGACACTATAGAATA** **CAGAAACGGATGGGAGTGC**	**GTACGACTCACTATAGGGA** **TATCAAGTGCAAGATCCCAATGAT**	**122**	***M***
**AIV-H5**	**AGGTGACACTATAGAATA** **CTTCAGGCATCAAAATGCACA**	**GTACGACTCACTATAGGGA** **TAGTTTGTTCATTTCTGAGTCGGTC**	**285**	***HA***
**AIV-H7**	**AGGTGACACTATAGAATA** **AATGGGGCHTTCATAGCTCC**	**GTACGACTCACTATAGGGA** **TGATAGCARTCRCCTTCACAA**	**144**	***HA***
**AIV-H9**	**AGGTGACACTATAGAATA** **ACAACAAGTGTGACAACAGAAGA**	**GTACGACTCACTATAGGGA** **TCTTCCGTGGCTCTCTCC**	**237**	***HA***
**DHV**	**AGGTGACACTATAGAATA** **TCTTCGTTGTGAAACGGATTACC**	**GTACGACTCACTATAGGGA** **TGCCTGGACAGATDTGTGCCTACT**	**133**	***5***′ ***UTR***
**DTMUV**	**AGGTGACACTATAGAATA** **ATGGACAGGGTCATCAGCGG**	**GTACGACTCACTATAGGGA** **GAATRGCTCCYGCCAATGCT**	**176**	***E***
**EDSV**	**AGGTGACACTATAGAATA** **AATCGGCAACTCAAGACATC**	**GTACGACTCACTATAGGGA** **CCCATTCATAAACAGGATTC**	**208**	***Penton***
**DEV**	**AGGTGACACTATAGAATA** **GGGAGGAGCAAACAAAGA**	**GTACGACTCACTATAGGGA** **ATCGCAAATTCCATCACATA**	**150**	***UL6***
**NDV**	**AGGTGACACTATAGAATA** **GTRGCAGCAAGRACAAGG**	**GTACGACTCACTATAGGGA** **CATATCYGCATACATCAA**	**196**	***L***
**DuCV**	**AGGTGACACTATAGAATA** **TGCKCCAAAGAGTCGACATA**	**GTACGACTCACTATAGGGA** **CAAAYGCATAACGGCTCTTTCC**	**300**	***Red***
**MDRV**	**AGGTGACACTATAGAATA** **CAGTTGAGCCGGAYGGTAATT**	**GTACGACTCACTATAGGGA** **ACTCGGTTGGTGTTAGTVGCVTAGAA**	**219**	***S1***
**MDPV**	**AGGTGACACTATAGAATA** **CTTTCAGGCTACATCTTCAA**	**GTACGACTCACTATAGGGA** **AATTCTCTTTTCACCCATCC**	**253**	***VP1***
**Universal tag sequences Tag-F:** **AGGTGACACTATAGAATA** **Tag-R:** **GTACGACTCACTATAGGGA**				

Universal tag sequences are underlined. Bold type indicates degenerate sites

Abbreviations: *M* A or C, *R* A or G, *W* A or T, *S* G or C, *Y* C or T, *K* G or T, *V* A, G, or C, *H* A, C, or T, *D* A, G, or T; *B* G, C, or T

Twelve specific genes from the eleven duck pathogens were amplified using the primers listed in Table 2. Plasmid-encoding genes from DEV (AV1221), EDSV (GEV), MDPV (GX-5) and DuCV (GX1006) were prepared and quantified according to Xie (26, 27). Plasmids encoding genes from AIV (H5N1 AIV Re-1, H7N2 AIV Duck/HK/47/76, and H9N6/Duck/HK/147/77), DHV (AV2111), DTMUV (GX201301), NDV (GX1/00) and MDRV (NM1) were used to produce ssRNA via in vitro transcription using a High-Yield Transcription Kit (Thermo Scientific, USA). The copy numbers of the ssRNAs for AIV (H5N1 AIV Re-1, H7N2 AIV Duck/HK/47/76, H9N6/Duck/HK/147/77), DHV (AV2111), DTMUV (GX201301), NDV (GX1/00) and MDRV (NM1) were calculated as described previously [23, 25].

### Setup of the GeXP assay and the reaction procedure

The reaction system was created using the GeXP Start-up Kit in a total volume of 20 μl containing 4 μl of Genome LabTM GeXP Start Kit 5 × PCR Buffer, 4 μl of MgCl_2_ (25 μM)_,_ 2 μl of mixed primers (containing 20–100 nmol/l of 12 pairs of gene-specific chimeric primers), 1.4 μl of JumpStart Taq DNA polymerase, and 0.5 pg–0.5 ng of cDNA or DNA template. Nuclease-free water was then added to the PCR reaction to achieve a final volume of 20 μl. Three optimized PCR amplification steps were performed according to the temperature-switch PCR (TSP) strategy [22]: step 1 was carried out with gene-specific sequences of chimeric forward and reverse primers (10 cycles of 30 s at 95 °C, 30 s at 55 °C, and 30 s at 72 °C); step 2 was carried out predominantly with chimeric forward and reverse primers (10 cycles of 30 s at 95 °C, 30 s at 63 °C, and 30 s at 72 °C); and step 3 was carried out predominantly with universal forward and reverse primers (20 cycles of 30 s at 95 °C, 30 s at 50 °C, and 30 s at 72 °C). The reactions were held at 4 °C after the amplification cycles.

### Separation by capillary electrophoresis and fragment analysis

After amplification, 1 μl of the PCR product was added to 20 μl of sample loading solution along with 0.16 μl of DNA Size Standard-400 (Genome Lab GeXP Start Kit Beckman Coulter), following protocols described previously [19]. After amplified DNA amplicons were separated, the data were imported into the analysis module of ExpressProfiler software as a tab-delimited file for subsequent analyses.

### Evaluating the specificity of the GeXP assay

cDNA and DNA templates were used for the GeXP-mono PCR assay and the GeXP-multiplex PCR assay. The GeXP-mono PCR assay was performed using a single template along with each pair of chimeric primers to determine the size of the amplification products for each target gene. The specificity of the GeXP-multiplex PCR assay was tested for each individual viral target gene, and the assay was performed using a single template. The mono-GeXP PCR assay and GeXP-multiplex PCR assay were developed using the reaction system and procedure described in Material and Methods Section 2.3.

### Evaluating the accuracy of the GeXP assay

The reaction system and procedures described above were used for the validation of GeXP-multiplex PCR for the detection of the twelve viruses. cDNA/DNA hybrids of all twelve duck viral disease agents were used as templates along with a primer mixture (0.2 μl). PCR product separation and detection were performed on a Genome Lab GeXP Genetic Analysis System (Beckman Coulter) via capillary electrophoresis, following the protocols described previously in Materials and Methods 2.3.

After the amplified fragments were separated, the peaks were initially analyzed using the fragment analysis module of the GeXP system software and matched to the appropriate genes. The peak height for each gene was reported in the electropherogram.

To emulate mixed infections, we randomly chose duck pathogens and extracted their DNA and RNA. RNA viruses were transcribed into cDNA as described above. DNA and cDNA were mixed together in equal concentrations to serve as templates for the optimized GeXP-multiplex PCR assay. To simulate the detection of a clinically mixed infection by GeXP, random cDNAs from eleven viruses or DNA samples from three to five viruses were mixed, and two groups were established. Group 1 consisted of a mixture of cDNAs from three avian influenza viruses (AIV-H5, AIV-H7 and AIV-H9) per template, while group 2 consisted of cDNA/DNA mixtures of DEV, DTMUV, NDV, EDSV and AIV-H5 viruses per template. A mixture of primers (0.2 μl) was added, and the remainder of the procedure was performed as described in Materials and Methods.

### Evaluating the sensitivity of the GeXP assay

The sensitivity of the GeXP-multiplex assay for each type of duck virus was examined using serial 10-fold dilutions of the plasmids (DEV, EDSV, MDPV, and DuCV) and ssRNAs obtained via in vitro transcription (AIV-5, AIV-7, AIV-9, DHV, DTMUV, NDV, and MDRV), either separately or with the twelve samples mixed together (10^6^ to 10^1^ copies/μl). The assays were performed in triplicate. Plasmid or ssRNA mixtures were used to test the detection limit when all twelve duck virus genotypes were present. PCR was performed using the same experimental conditions described above for the GeXP-multiplex PCR assay, and the detection limit of the GeXP-PCR was determined based on the most dilute template that yielded a positive result. After amplification, 2 μl of each Cy5-labeled PCR product was separated via GeXP capillary electrophoresis and detected by fluorescence spectrophotometry.

### Interference assay

Because the presence of other templates in high quantities could alter the efficiency of GeXP-multiplex PCR amplification, different amounts of the templates (10^3^ to 10^7^ copies) were selected at random, mixed and tested in the GeXP-multiplex PCR assay. The results were compared with those of the single-template GeXP-multiplex PCR assay.

### Evaluation of the GeXP-PCR assay using clinical samples

A total of 150 archived clinical specimens were collected from ducks in Guangxi, China, between July, 2012 and November 2013. RNA and DNA from the 150 specimens were then extracted using the MiniBEST Viral RNA/DNA Extraction Kit (Takara, Dalian, China) and a TIANamp Bacteria DNA Kit (Tiangen, Beijing, China). Genomic RNA from all samples was transcribed into cDNA as described above, and cDNA samples were analyzed using the optimized GeXP-multiplex PCR assay in addition to conventional simplex PCR methods with same primers as the GeXP assay. The obtained positive samples were send to a company (BGI, China) for sequencing.

## Results

### Specificity of the GeXP-multiplex PCR assay

The DNA and cDNA from eleven duck pathogens were used as templates to evaluate the specificity of each pair of gene-specific primers. In mono-GeXP PCR assays, the AIV universal primers that we designed could amplify the target *M* genes of all AIV serotypes; however, each pair of pathogen-specific primers amplified only the corresponding genes from the targeted pathogens without cross-amplification. The expected amplification peaks were observed in the GeXP-multiplex PCR assay (Table 1). No specific amplification peaks were observed when duck *Escherichia coli*, duck *Salmonella,* duck *Staphylococcus aureus, Pasteurella multocida,* infectious bronchitis virus, or *Mycoplasma gallisepticum* were tested (Table 1).

**Fig. 1:**
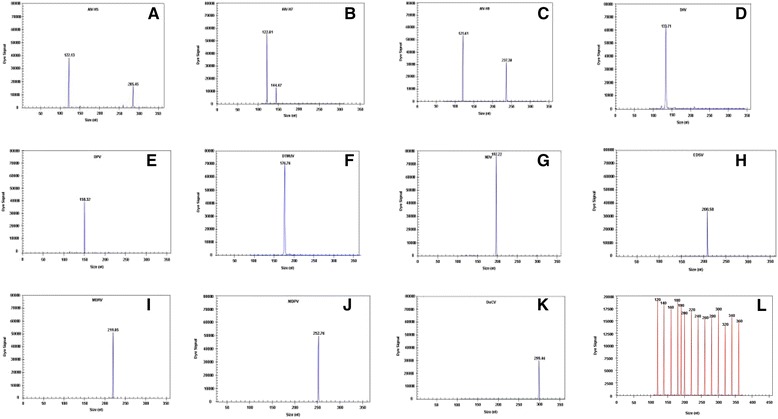
Specificity analyses of the GeXP-PCR assay. The Y-axis indicates the dye signal in A.U., and the X-axis indicates the actual PCR product size. **a**–**k** show the results of the amplification of AIV-H5, AIV-H7, AIV-H9, DHV, DEV, DTMUV, NDV, EDSV, MDRV, MDPV, and DuCV, respectively. Nuclease-free water was used as the negative control (**l**). The red peaks indicate the DNA size standard

The GeXP-multiplex PCR assay used twelve pairs of primers to detect eleven duck viruses, and specific amplification peaks were observed (Fig. 1). Specific amplification peaks corresponding to the *M* gene of AIV were observed upon testing H1N3, H1N1, H2N3, H3N6, H4N5, H6N8, H8N4, H10N3, H11N3, H12N5 and H13N5.

### Sensitivity of the GeXP assay

**Fig. 2:**
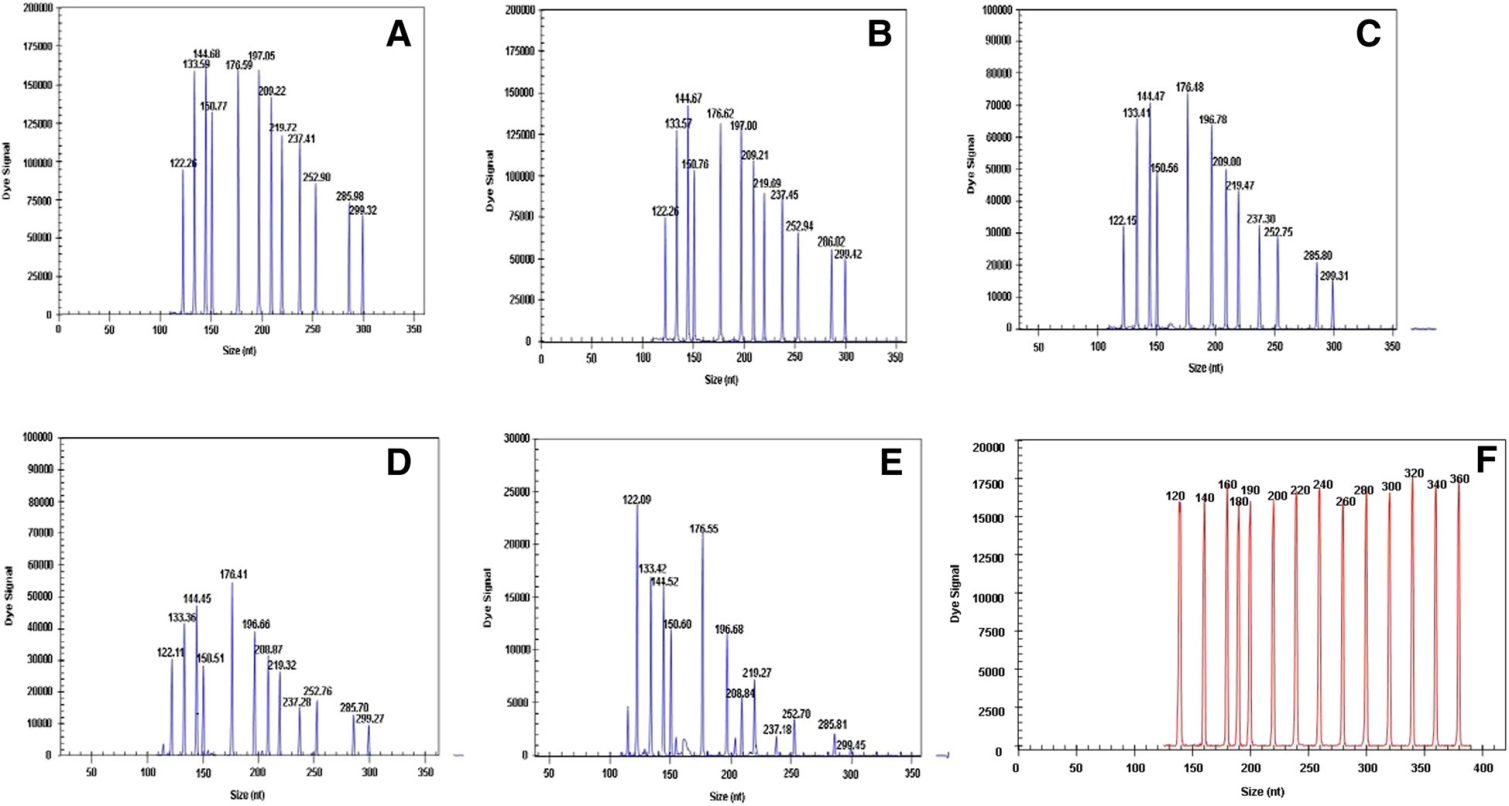
Sensitivity of the GeXP-PCR assay. Serial 10-fold dilutions of plasmids containing the 12 duck virus types were prepared and amplified in the GeXP-PCR assay using equal amounts of template: 10^6^ (**a**), 10^5^ (**b**), 10^4^ (**c**), 10^3^ (**d**) and 10^2^ (**e**) copies per reaction in the GeXP-PCR assay. The viral targets from left to right are as follow: AIV-M, DHV, AIV-H7, DEV, DTMUV, NDV, EDSV, MDRV, AIV-H9, MDPV, AIV-H5, and DuCV. Nuclease-free water was used as the negative control (**f**)

The GeXP-PCR assay was capable of detecting as few as 10-100 copies of each of the twelve recombinant plasmids of duck viruses (data not shown) and as few as 10^3^ copies when all of the twelve pre-mixed duck virus targets were present in a mixture (Fig. 2). Typically, a reaction is considered positive when the A.U. value is over 2000 by default [22], and 10 duck viruses were detected at a concentration of 10^2^ copies per reaction (DuCV being the exception). The reactions were repeated three times at each template concentration, and similar results were obtained.

### Artificial mixture

**Fig. 3:**
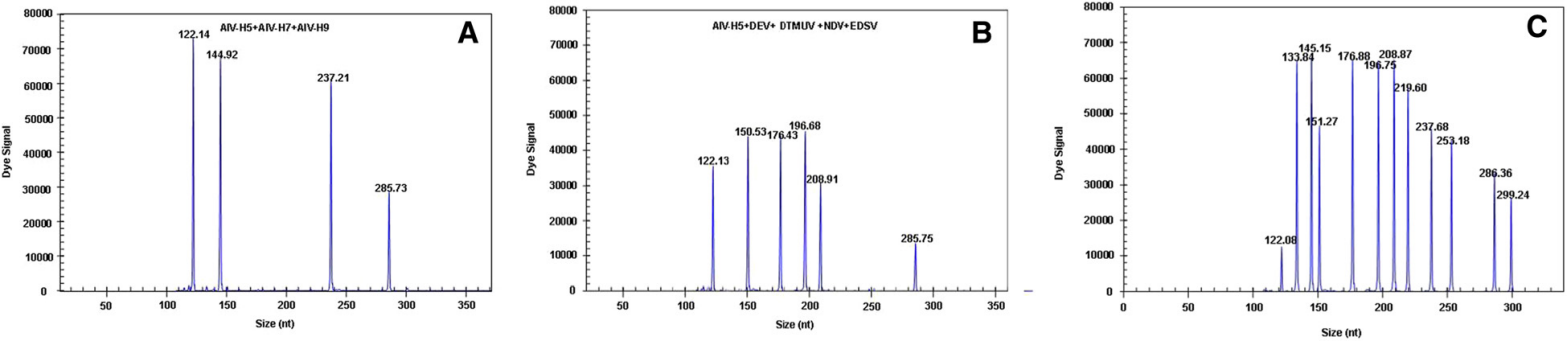
GeXP-multiplex PCR detection of mixed pathogen templates. The GeXP-multiplex assay was carried out with mixed templates and mixed primers for AIV-H5, AIV-H7 and AIV-H9 (**a**); AIV-H5, DEV, DTUMV, NDV and EDSV (**b**); or all eleven viruses (**c**)

The cDNA and DNA from the duck pathogens were mixed together to test the ability of the GeXP-multiplex PCR assay to differentiate among them, and the appropriate specific amplification peaks and universal amplification peaks were observed (Table 1; Fig. 3a, b). When AIV-H5, AIV-H7and AIV-H9 were mixed together and used in the GeXP-multiplex PCR assay, 3 specific amplification peaks (AIV-H7, 144.92 bp; AIV-H9, 237.21 bp; AIV-H5, 285.73 bp) and one universal amplification peak (AIV *M* gene, 122.14 bp) were observed (Fig. 3a). When AIV-H5, DEV, DTMUV, NDV and EDSV were mixed together and used in the GeXP-multiplex PCR assay, there were six specific amplification peaks (Fig. 3b). When the cDNA and DNA of all eleven pathogens were mixed and used in the GeXP-multiplex PCR assay, eleven specific amplification peaks were observed (Table 1; Fig. 3c).

### Interference assay

**Fig. 4:**
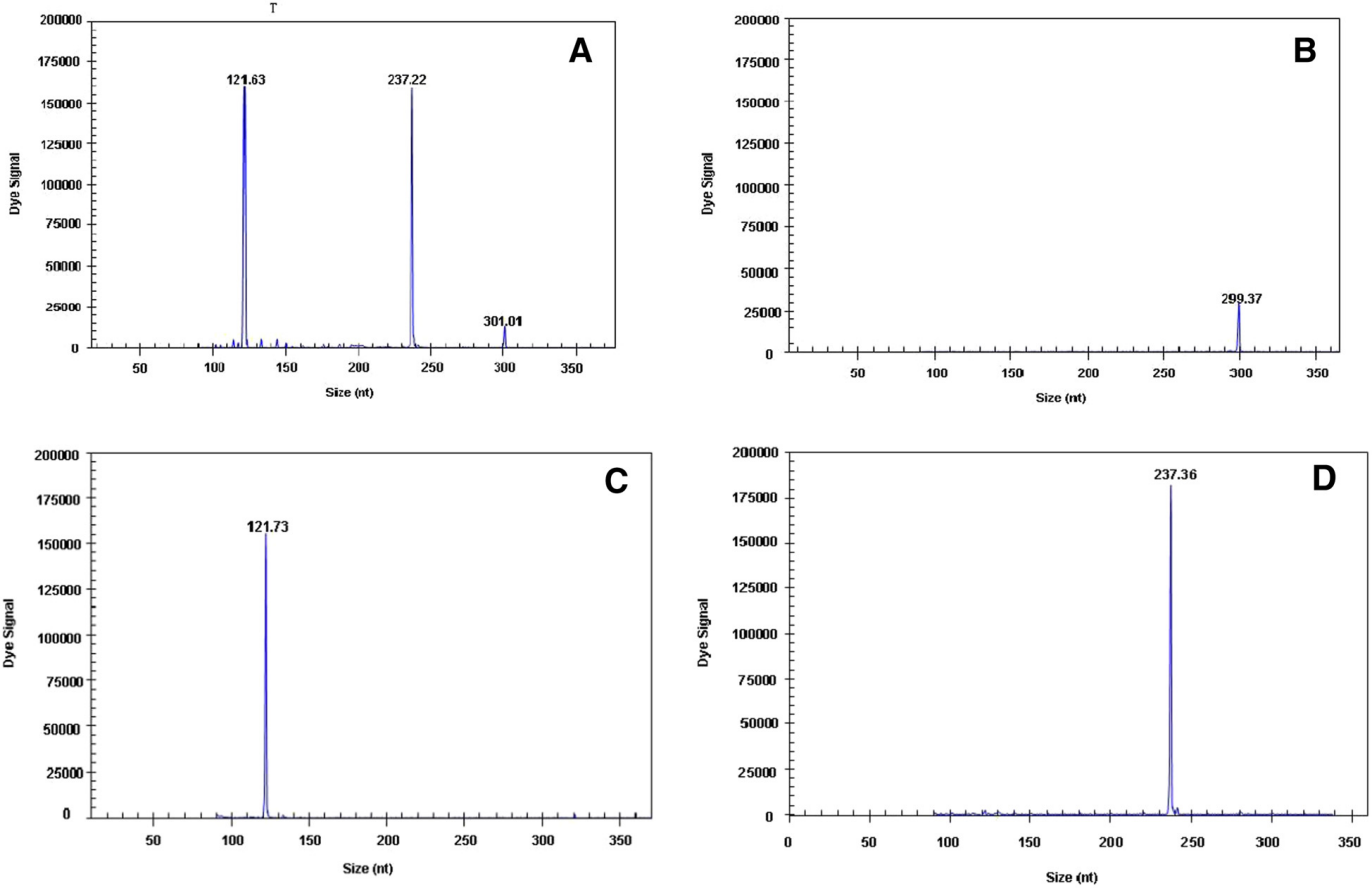
GeXP-multiplex PCR interference assay. GeXP-multiplex PCR was carried out with the following templates: AIV-M + AIV-H9 + DuCV (**a**), DuCV (**b**), AIV-M (**c**), and AIV-H9 (**d**)

Three specific amplification peaks were observed when three different templates (one template with 10^3^ copies and two templates with 10^7^ copies) were tested using the GeXP-multiplex PCR assay. Additionally, the peak value of a single template was similar to that of a mixed template. For example, three specific amplification peaks were observed when three different templates (10^3^ copies of DuCV and 10^7^copies of AIV-M and AIV-H9) were tested using the GeXP-multiplex PCR assay (Fig. 4), and the peak values for AIV-M, AIV-H9 and for DuCV were the same regardless of whether a single template (AIV-M and AIV-H9 or DuCV) or a mixed template (AIV-M + AIV-H9 + DuCV) was utilized. The results of these experiments showed that mixed infections can be detected by GeXP-multiplex PCR with only minimal interference.

### Assay of field samples using GeXP-multiplex PCR

A total of 150 clinical samples were assayed using the optimized GeXP-multiplex PCR method in addition to the conventional simplex PCR method. The positive and negative results obtained with the different methods are shown in Table 3. The degree of consistency between and the GeXP assay and conventional PCR methods is shown in Table 4. The kappa values as a measure of agreement for the GeXP assay and conventional PCR methods were as follow: K = 0.908, K > 0; u = 28.375, u > 1.96, *P* < 0.05. The two experimental results appear to have a high degree of consistency. All positive specimens in the GeXP assay and conventional methods were identified via sequencing (BGI, China) to be true positive samples.

**Table 3:** Analysis of clinical samples using the GeXP assay and conventional PCR methods

Clinical samples	No. of GeXP assay results		No. of conventional PCR method results	
	Positive	Negative	Positive	Negative
H9 subtypes of AIV	20	130	18	132
DHV	5	145	5	145
DEV	1	149	1	149
DTMUV	5	145	5	145
NDV	10	140	9	141
EDSV	3	142	3	147
MDRV	1	149	1	149
MDPV	4	146	4	146
DuCV	40	110	35	115
Total	89	61	81	69

**Table 4:** Comparison of clinical samples using the GeXP assay and conventional PCR methods

GeXP assay results	Conventional PCR methods results		Total
	+	−	
+	81	8	89
−	0	61	61
Total	81	69	150

## Discussion

AIV-H5, AIV-H7, AIV-H9, DHV, DEV, DTMUV, NDV, EDSV, MDRV, MDPV, and DuCV are the eleven types of viruses that cause infectious diseases in duck. In this study, we developed a GeXP method for the simultaneous detection of these eleven duck viruses. Twelve pairs of specific primers were designed according to the conserved sequences of the genes from each pathogen; these sequences were obtained from GenBank. The GeXP genetic analysis system is a multitarget, high-throughput detection platform, and its application in the differential detection of nine avian respiratory pathogens was reported recently by our laboratory [23, 26] .

The amplification specificity and accuracy of each primer set in GeXP-multiplex PCR was verified by the inspection of samples containing a single virus or a mixed population and positive clinical samples (including mixed infections, single infections and infections with more than two types of pathogen) were used to further verify the method. The evaluation of sensitivity using recombinant plasmids for each duck virus revealed that all twelve of the hybrid templates were detected simultaneously when they were present at a concentration of 10^3^ copies per reaction, indicating the high sensitivity of the GeXP-PCR assay. One hundred and fifty specimens were analyzed by the GeXP-PCR assay (89 were positive) and conventional PCR methods (81 were positive). Additionally, 8 clinical samples that were negative based on conventional single PCR but positive in the GeXP assay were confirmed later by sequencing to be true positives (data not shown). Compared with the results of conventional PCR method, the GeXP-multiplex PCR method was more sensitive and accurate.

In recent years, multiplex PCR and multiplex fluorescence real-time quantitative PCR techniques have been widely used for the detection of mixed infections with multiple pathogens [27]. Multiple PCR involves the use of several primer pairs in one reaction system at the same time. Thus competition occurs during amplification as the primer pairs interfere with each other. The probability of forming complex primer dimers increases, and the sensitivity decrease. Additionally, multiplex PCR products are typically observed by agarose gel electrophoresis, which is not an optimal method for distinguishing bands that are less than 100 bp in length. Furthermore, the probes used in multiplex fluorescence real-time quantitative PCR are conjugated to fluorophores that emit light at different wavelengths. General PCR methods, such as conventional multiplex PCR and multiplex real-time PCR [28–32], can only detect 2 to 6 types of pathogens; thus, these methods are not ideal for the rapid high-throughput detection of gene expression or analysis of multiple pathogens. Quantitative GeXP expression analysis combines multiplex PCR and capillary electrophoresis, employing fluorescently labeled universal primers and specific primer combinations to trigger multiple amplifications. Using this technique, multiple genes can be amplified using 1 pair of universal primers. The amplification efficiency of each template is consistent, and the amplification efficiency of each primer pair is not affected. Thus, high-throughput detection and identification of multiple pathogens can be achieved using this method.

Two other distinct advantages of this GeXP-multiplex PCR method are the short assay time and the low cost [33, 34]. The entire reaction can be completed in one tube within 2.5 h followed by capillary electrophoresis separation. In addition, two 96-well plates can be placed in parallel in a GeXP analyzer at the same time to further increase the throughput of this method.

The findings described here may lead to increased utilization of these gene-based tests for the routine diagnosis of viral infection. In addition, some of the viruses we detected in the assay may not be pathogenic in animals or could be live virus vaccine strains found in the animal herds. However, the data may still be valuable for epidemiological studies. To make a definite diagnosis, we must consider the clinical symptoms and whether live vaccine was recently injected. Furthermore, we have found that the GeXP analyzer is an efficient and easy-to-use tool; thus it may be a useful new technology for the identification of new biomarkers for diagnosing viral diseases in ducks.

## Conclusions

This study has demonstrated that the GeXP-multiplex PCR assay is a rapid method with high sensitivity and specificity for the identification of three very important avian influenza subtypes (H5, H7, H9) in addition to eight other duck pathogens. This method may therefore be adopted for the molecular epidemiologic surveillance of these duck pathogens.

## Abbreviations

AIV: Avian influenza virus
DHV: Duck hepatitis virus
DTMUV: Duck tembusu virus
EDSV: Egg drop syndrome virus
DEV: Duck enteritis virus
NDV: NEWCASTLE disease virus
DuCV: Duck circovirus
MDRV: Muscovy duck reovirus
MDPV: Muscovy duck parvovirus
GeXP: Genome Lab Gene Expression Profiler
IACUC: Institutional Animal Care and Use Committee
TSP: Temperature-switch PCR
